# Milk thistle seed extract protects rat C6 astroglial cells from acute cocaine toxicity

**DOI:** 10.3892/mmr.2014.2524

**Published:** 2014-08-28

**Authors:** RAMESH B. BADISA, CHERYL A. FITCH-PYE, MARYAM AGHARAHIMI, DONALD E. PALM, LEKAN M. LATINWO, CARL B. GOODMAN

**Affiliations:** 1 College of Pharmacy and Pharmaceutical Sciences, Florida A&M University, Tallahassee, FL 32307, USA; 2 Department of Biological Science, Florida State University, Tallahassee, FL 32310, USA; 3 Department of Biological Science, Florida A&M University, Tallahassee, FL 32307, USA

**Keywords:** acute cocaine, C6 astroglial cells, *Silybum marianum*, pretreatment, glutathione

## Abstract

Cocaine is a powerful addictive drug, widely abused in most Western countries. It easily reaches various domains within and outside of the central nervous system (CNS), and triggers varying levels of cellular toxicity. No pharmacological treatment is available to alleviate cocaine-induced toxicity in the cells without side-effects. Here, we discerned the role of milk thistle (MT) seed extract against cocaine toxicity. First, we investigated acute cytotoxicity induced by treatment with 2, 3 and 4 mM cocaine for 1 h in astroglial, liver and kidney cells *in vitro*, and then in living shrimp larvae *in vivo*. We showed that astroglial cells are more sensitive to cocaine than liver, kidney cells or larvae. Cocaine exposure disrupted the general architecture of astroglial cells, induced vacuolation, decreased cell viability, and depleted the glutathione (GSH) level. These changes may represent the underlying pathology of cocaine in the astrocytes. By contrast, MT pretreatment (200 μg/ml) for 30 min sustained the cell morphological features and increased both cell viability and the GSH level. Besides its protective effects, the MT extract was revealed to be non-toxic to astroglial cells, and displayed high free-radical scavenging activity. The results from this study suggest that enhanced GSH level underlies cell protection, and indicate that compounds that promote GSH synthesis in the cells may be beneficial against cocaine toxicity.

## Introduction

Cocaine is one of the most powerful addictive drugs widely abused, mostly in Western countries. This drug is an alcaloid, naturally occurring in the *Erythroxylon* plants of the Erythroxylaceae family. In certain countries like Argentina, Bolivia, Columbia and Peru, certain species of the genus *Erythroxylon* are used as cash crops. Cocaine possesses two pharmacological actions: one as a local anesthetic and the other as a psychostimulant. The anesthetic property has been experienced by the people of different countries for several centuries by chewing the leaves of *Erythroxylon* plants to treat headaches, tooth aches and body pains. However, since repeated consumption of cocaine causes a state called ‘drug-dependence’ its possession or use or distribution is illegal in most countries.

Cocaine easily reaches various domains of the brain and vital organs outside of the central nervous system (CNS), such as liver, heart, and kidney, and triggers different levels of cellular toxicity. Current estimates indicate that approximately 1.4 million Americans regularly use cocaine (1). To date, there is no specific pharmacological medication approved by the Food and Drug Administration to treat cocaine toxicity (2). Several drugs that are originally designed for treatment of diseases have been tested in this context, but none has proved satisfactory in terms of outcome. For example, modafinil, antabuse and bromocriptine show numerous undesirable side-effects on healthy cells (3–6). The profound limitations of these drugs clearly highlight the need to investigate other compounds as potential therapeutic agents for the treatment of cocaine addiction.

Compounds that are relatively devoid of undesirable side-effects are highly desirable for use as therapeutic agents, and are of importance in pharmaceutical applications, since they address issues concerning the quality of life in general. It is widely believed that most herbal products have mild to no side-effects, and these products are often used as alternative medicine for several types of diseases and disorders. For centuries, herbal products have been used to treat different types of ailments. Recently, the use of herbal products has significantly increased, due to the fact that synthetic drugs have a high cost, a short shelf-life and severe side-effects. It is not surprising that >40% Americans currently use complementary medicine for its various health benefits (7). One of the herbal products used worldwide comes from the milk thistle (MT).

MT, or *Silybum marianum* (Linn.) Gaertneri, is a plant species of the Asteraceae/Compositae family. The fruits of MT have been used for a long time as a remedy for liver and gallbladder disorders (8). Results of preclinical studies have highlighted the benefits of using MT or its purified compounds without any adverse reactions (9–11). Silymarin was identified as the active constituent of MT that is responsible for these effects (8). In the USA, MT is commonly used as a dietary supplement. Given its protective effects on liver, we hypothesized that MT may play an important role against cocaine-induced toxicity in different cell types.

In the present study, we evaluated cocaine toxicity in different tissue cell lines, namely astroglial, liver, and kidney cells. Then, we investigated the protective role of MT against cocaine-induced toxicity. We specifically used MT seed powder, since MT seeds are recognized for their medicinal value. Silymarin, the active ingredient of MT, represents 80% of the seed powder. The short-term effects of cocaine and MT were also evaluated on living brine shrimp larvae, which have a relatively simple nervous system; these experiments further provided the opportunity to directly monitor the behavior of living shrimp larvae in the presence of cocaine.

## Materials and methods

### Materials

RPMI-1640 (cat. no. 15-040-CV, Mediatech, Inc. Manassas, VA, USA), fetal bovine serum (FBS), penicillin/streptomycin sulfate, amphotericin B, phosphate-buffered saline (PBS), and L-glutamine were purchased from Mediatech (Herndon, VA, USA). Cocaine hydrochloride (ecgonine methyl ester benzoate), crystal violet, 2,2-diphenyl-1-picrylhydrazyl, L-glutaraldehyde, 0.5 M ethylene diamine tetraacetic acid (EDTA) solution, 5,5-dithiobis-2-nitrobenzoic acid (DTNB), glutathione (GSH) reductase (cat. no., G-6004), N-acetyl-L-cysteine (NAC; ≥99% purity), nicotinamide adenosine dinucleotide phosphate (NADPH), 5-sulfosalicylic acid, and trypan blue were supplied by Sigma-Aldrich (St. Louis, MO, USA). The remaining chemicals used in this study were of analytical grade.

### Cell lines and animals

The CNS-derived C6 astroglial cell line CCL-107, the liver cell line CRL-1439, and the MDCK kidney cell line CCL-34 were obtained from the American Type Culture Collection (Rockville, MD, USA), and were maintained separately as monolayer cultures. Astroglial and liver cells were cultured as described earlier (12), while MDCK cells were grown in Dulbecco’s modified Eagle’s medium (DMEM) with 4,500 mg/l high glucose (cat. no. D-6429, Sigma-Aldrich, St. Louis, MO, USA). All media were supplemented with 2 mM L-glutamine, 10% (v/v) FBS, 100 U/ml penicillin, 100 μg/ml streptomycin and 0.25 μg/ml amphotericin B. Cells were grown in an incubator with humidified atmosphere containing 5% CO_2_, at 37°C. The brine shrimp cysts (*Artemia salina*) were obtained from a local pet store (Carol’s Critters, Tallahassee, FL, USA). The cysts were seeded for rehydration on the surface of artificial seawater, prepared with 1.9% salt mixture (Instant Ocean, Aquarium Systems, Inc., Mentor, OH, USA) in deionized water in a tank under constant illumination at room temperature (22–28°C) for 48 h.

### Treatments

Cocaine treatment was carried out in polystyrene, flat-bottom 96-well microtiter plates. Astroglial and liver cells were seeded at a starting density of 2×10^4^ cells/well in a final volume of 195 μl RPMI-1640 supplemented with 10% FBS. The cells were allowed to adhere overnight in the incubator. Kidney cells have the ability to form tight junctions, and were thus seeded at an initial density of 2×10^3^ cells/well in a final volume of 97.5 μl of DMEM supplemented with 10% FBS. However, prior to cocaine treatment of kidney cells, the DMEM was replaced with RPMI-1640 supplemented with 10% FBS so that media in all cultures remained the same. Cocaine treatment of astroglial and liver cells was performed on the day following cell seeding, while treatment of kidney cells started four days after seeding, in order to facilitate the formation of tight junctions. On the day of treatment, 1 M stock solution of cocaine was prepared in PBS immediately prior to the assay. From the stock solution, various working dilutions (80–160 mM) were prepared in PBS, and added to the cells in order to achieve final concentrations of 2, 3 and 4 mM cocaine. Addition of the cocaine solution was performed in a minimum volume (5 μl/well for astroglial and liver cells; 2.5 μl/well for kidney cells), to avoid changes in the pH of the medium. Untreated cells received an equal volume of PBS (5 or 2.5 μl/well) and served as the vehicle control. Cells with medium only (200 or 100 μl/well) served as an additional negative control. Treatments lasted 1 h and were performed at a 37°C, 5% CO_2_ incubator. The duration of cocaine treatment (1 h) was selected based on our earlier study (13).

### Viability, morphology and vacuolation

At the end of the 1-h incubation, cell viability was evaluated by a crystal violet dye uptake assay as in (14). For gross evaluation of morphological changes and vacuolation, the crystal violet (0.1%)-stained cells were observed under an inverted phase contrast IX-70 Olympus microscope (Olympus, Melville, NY, USA) with a ×40 objective, and pictures were acquired as described elsewhere (12). The neutral red dye uptake (0.05%) method was adopted to quantify the vacuoles in the cells, as described in (15).

### Antioxidant activity of MT

A known amount of MT dry powder (Paradise Herbs and Essentials, Inc., Huntington Beach, CA, USA) from capsules (crude extract from seeds, containing 80% silymarin) was dissolved in dimethyl sulfoxide (DMSO), to obtain a stock solution of 25 mg/ml. The antioxidant activity assay was carried out in eppendorf tubes without cells at different concentrations of MT (25, 50, 75 and 100 μg/ml), using ethanol as the solvent. NAC, a known antioxidant, was used as a positive control (1 mM, 0.163 mg/ml). The percentage of scavenging activity was calculated based on a method described earlier (16).

### Evaluation of MT cytotoxicity in astroglial cells

Experiments were performed in 96-well culture plates for astroglial cells, in a total volume of 195 μl RPMI-1640 medium supplemented with 10% FBS. From the MT stock solution, various working stocks (0.1 to 8 mg/ml) were prepared in the medium prior to treatment and added to the cells in a minimum volume of 5 μl/well, to achieve final concentrations of 25 to 200 μg/ml. Untreated cells received an equal volume of DMSO (0.8% final) and served as the vehicle control. Cells in medium only (200 μl/well) served as an additional negative control. Treatment lasted 1 h and was performed at a 37°C, 5% CO_2_ incubator. Cell viability was assessed as previously described (14).

### Pretreatment of astroglial cells with MT

Experiments were performed in 96-well culture plates for astroglial cells, in a total volume of 190 μl RPMI-1640 medium supplemented with 10% FBS. The cells were pretreated with 200 μg/ml MT (5 μl/well, final DMSO, 0.8%) for 30 min, followed by co-treatment with cocaine (5 μl/well, 2–4 mM final) for 1 h. Cells containing 0.8% DMSO in the medium, and cells with medium only served as the negative controls. At the end of the incubation, cell viability was measured as described earlier (14), and the GSH level was assessed as described below.

### Total GSH level in astroglial cells

Cellular glutathione levels were assayed according to the method described in Smith *et al* (17). Briefly, following treatment with three different concentrations of cocaine (2–4 mM) in RPMI-1640 medium for 1 h in 96-well microtiter plates, the cells were deproteinized with 2% 5-sulfosalicylic acid (10 μl/well) for 30 min at 37°C, followed by addition of 90 μl of reaction mixture, containing 0.416 mM sodium EDTA, 0.416 mM NADPH, 0.835 mM DTNB, glutathione reductase (0.825 units) and 0.083 mM sodium phosphate buffer, pH 7.5. The cells were incubated with the mixture for 30 min at 37°C. The absorbance (Abs) was measured at 412 nm on a MQX200; microplate reader (Bio-Tek Instruments Inc., Winooski, VT, USA).

### In vivo brine shrimp lethality assay

The brine shrimp larvae lethality assay was performed as described by Meyer *et al* (18) and with modification suggested by McLaughlin (19). The assay was carried out in triplicate vials with ten larvae each, and with fixed doses of MT (200 μg/ml), or various doses of cocaine (2, 3 and 4 mM). Untreated shrimp larvae in artificial seawater, or DMSO (0.8%)-exposed larvae in artificial seawater served as controls. The treatment lasted 1 h at room temperature, under constant light.

### Statistical analysis

The experimental results were expressed as the mean ± standard error of the mean (SEM). Data were analyzed by a one-way analysis of variance (ANOVA), followed by Dunnett’s multiple comparison tests, using the GraphPad Prism software, version 5.00 (GraphPad Software, San Diego, CA, USA). The values of the lethal concentration needed to kill 50% of the cells (LC_50_) and of the median effective dose (ED_50_), were determined from the graphs based on a method described earlier (20).

## Results

### Dose-dependent toxicity of cocaine

**Figure 1:**
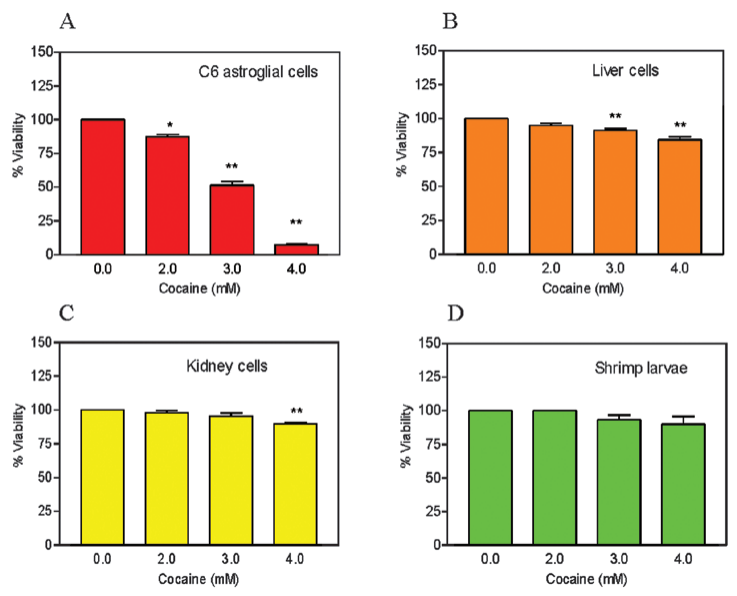
Effect of cocaine on viability. (A) C6 astroglial, (B) liver and (C) kidney cells were treated with various concentrations of cocaine for 1 h in 96-well plates, and the viability was assessed by the crystal violet dye uptake method. (D) Living shrimp larvae were counted in each vial at the end of a 1-h cocaine treatment. Data are expressed as mean ± standard error of the mean (SEM), ^*^P<0.05 or ^**^P<0.01, compared to the control (0 μM cocaine).

The toxic effect of cocaine was evaluated *in vitro* following 1 h of exposure of C6 astroglial, liver, and kidney cells to 2, 3, and 4 mM of the drug. The astroglial cells were more sensitive to cocaine (Fig. 1A) than liver and kidney cells. The percentage of viability of astroglial cells significantly decreased (P<0.05, n=3) with the increasing cocaine dose, an observation consistent with an earlier study on these cells (14). Compared to the control (100% viability), the average astroglial cell viability (± SEM) at 2, 3, and 4 mM of cocaine was 88±1.1, 51±2.2, and 7±1%, respectively. The cocaine LC_50_ was 3.0 mM in these cells. Notably, cocaine treatment did not markedly alter the viability of liver and kidney cells (Fig. 1B and C). The cocaine LC_50_ in these cell lines was >4.0 mM.

We then used living brine shrimp larvae as a simple *in vivo* model to evaluate the effects of treatment with different concentrations of cocaine (2, 3 and 4 mM) for 1 h. All larvae in the control vials were alive and active, swimming in all directions. Treatment with cocaine for 1 h did not cause significant shrimp death, nor did it affect their activity. All larvae were active and resembled the controls (P>0.05). The ED_50_ was >4 mM (Fig. 1D).

### Antioxidant activity of MT

**Figure 2:**
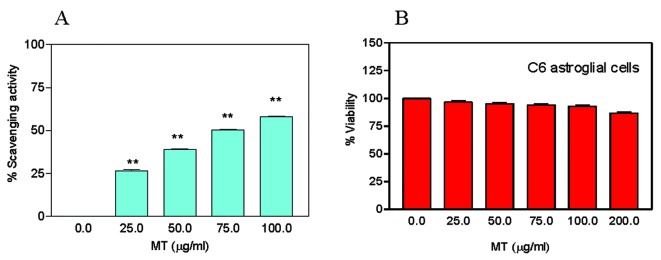
Measurement of antioxidant and cytotoxic activities of milk thistle (MT) extract. (A) Various concentrations of MT dissolved in ethanol in 1-ml eppendorf tubes without cells, were incubated in 0.1 mM of the free radical 2,2-diphenyl-1-picrylhydrazyl for 30 min. (B) C6 astroglial cells were treated with different concentrations of MT for 1 h, and viability was assessed using the crystal violet dye (0.1%) uptake method. Data are expressed as means ± standard error of the mean (SEM), ^**^P<0.01, compared to the control (0 μM MT).

Prior to evaluating the potential role of MT in alleviating the cocaine-induced toxicity, we determined its antioxidant activity. It has been previously reported that, although most plant extracts exhibit antioxidant activity, different extraction procedures can affect this activity, with variations ranging from 53 to 173 μg/ml (21,22). MT exhibited a dose-dependant increase in the free radical scavenging activity (Fig. 2A). Compared to the control, the average activity (± SEM) at 25, 50, 75, and 100 μg/ml was 26.5±0.6, 38.9±0.4, 50.4±0.3, and 58.1±0.5%, respectively. The MT ED_50_ free radical scavenging activity was evaluated at ~75 μg/ml. NAC (7.66 μg/ml, MW, 163.19), used as a positive control in this study, had an ED_50_ of 0.047 mM. These results demonstrate that MT has a relatively high antioxidant activity.

### MT is not cytotoxic

We sought to determine the highest non-toxic dose of MT required to alleviate cocaine toxicity in astroglial cells. For this purpose, we treated the cells with 25, 50, 75, 100, and 200 μg/ml of MT for 1 h. MT did not cause significant death in astroglial cells (P>0.05) at any concentration (Fig. 2B). The estimated LC_50_ was >200 μg/ml, therefore, we used this concentration in the following experiments. Treatment with 200 μg/ml of MT for 1 h did not cause death in the larvae either, as determined by the brine shrimp assay (data not shown).

### MT pretreatment protects from cocaine toxicity, morphological alterations and vacuolation

**Figure 3:**
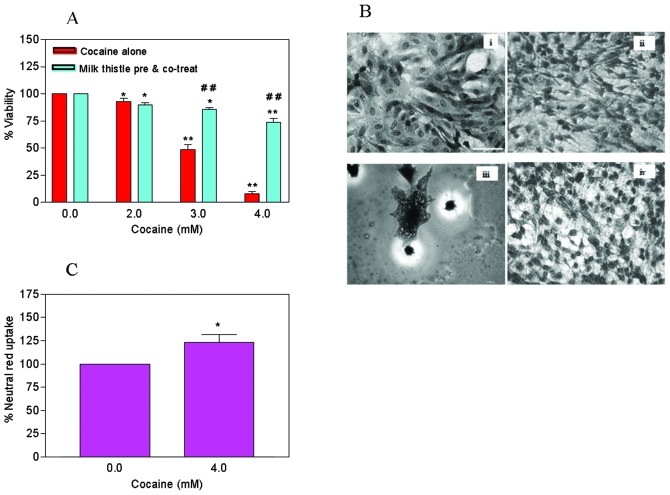
Attenuation of cocaine-induced toxicity by milk thistle (MT) extract in C6 astroglial cells. Cells were pretreated with 200 μg/ml MT for 30 min, followed by co-treatment with 2, 3 or 4 mM cocaine for 1 h. (A) Cell viability was evaluated with the crystal violet (0.1%) dye uptake assay and (B) images of control (i), MT (ii), 4 mM cocaine (iii) and MT-pretreated and 4 mM cocaine co-treated cells (iv) were acquired under an inverted phase contrast 1X-70 Olympus microscope with a ×40 objective. Scale bar, 50 μm. (C) Vacuolation in control and 4 mM cocaine-treated cells was quantified with the neutral red dye (0.05%) uptake assay. Data are expressed as mean ± standard error of the mean (SEM), ^*^P<0.05 or ^**^P<0.01, compared to the respective controls; ^##^P<0.01, cocaine treatment alone vs. MT-pretreated and cocaine co-treated group.

The astroglial cells were pretreated with 200 μg/ml of MT for 30 min, followed by co-treatment with 2, 3, or 4 mM cocaine for 1 h. The viability of MT-pretreated, cocaine co-treated cells was significantly increased (P<0.01) compared to the cells treated with cocaine alone (Fig. 3A). The average cell viability (± SEM) following MT pretreatment was increased to 86±1.7 and 74±3.7%, respectively, compared to non MT-pretreated cells, treated with 3 and 4 mM of cocaine (50±2.6 and 8±1.1%, respectively). These results clearly indicated that MT provides important cell protection against cocaine toxicity.

Furthermore, microscopic observations of crystal violet (0.1%)-stained cells revealed that, in comparison to the shape of untreated control cells (Fig. 3B-i), there was no significant morphological difference in the MT-pretreated control cells (Fig. 3B-ii) or in the MT-pretreated and cocaine co-treated cells (Fig. 3B-iv). Morphological observations also indicated that in the absence of MT pretreatment, cocaine elicited the formation of vacuoles in the cytoplasm (Fig. 3B-iii and C). However, pretreatment with 200 μg/ml MT not only prevented vacuole formation (Fig. 3B-iv), but also maintained the morphological features of the cells (Fig. 3B-i). These results suggest that astroglial cells are highly sensitive to cocaine, while MT pretreatment allows to maintain cell morphology in cocaine-treated cells.

### MT pretreatment increases the intracellular GSH level

**Figure 4:**
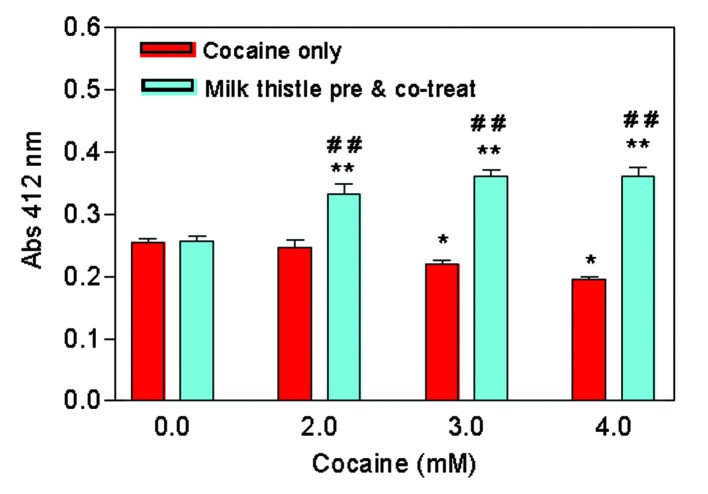
Effect of milk thistle (MT) extract on the total glutathione level. C6 astroglial cells were pretreated with 200 μg/ml MT for 30 min, followed by co-treatment with cocaine for 1 h. Data are expressed as mean ± standard error of the mean (SEM). ^*^P<0.05 or ^**^P<0.01, compared to the respective controls; ^##^P<0.01, cocaine treatment alone vs. MT-pretreated and cocaine co-treated group. Abs, absorbance.

We evaluated the role of MT on the cellular GSH level. Since we used the glutathione reductase enzyme in the assay, our glutathione measurements represent the total glutathione level, i.e., oxidized and reduced. The GSH level remained the same in MT-pretreated and control cells, treated with 0 mM of cocaine (Fig. 4). In the absence of pretreatment with MT, the GSH level was significantly decreased (P<0.01) to 97.3±3.8, 87.1±1.8, and 76.8±1.5%, at 2, 3, and 4 mM of cocaine, respectively, compared to the level of the control (Fig. 4). Pretreatment of cells with MT markedly (>25%) increased the GSH level at all doses compared to the cells treated with cocaine alone (Fig. 4). These data indicate that MT pretreatment increases the total GSH level in cocaine-treated cells.

## Discussion

The cocaine concentrations used in this study were similar to those applied in other *in vitro* experiments, reported earlier (12,23–25), where the LC_50_ or EC50 values were in the millimolar range (>3 mM). Under our experimental conditions, the cocaine LC_50_ in C6 astroglial cells was ~3 mM at 1 h exposure. This value is closer to that of a recent report (13). It is notable that cocaine was not prominently toxic to liver and kidney cells or to living shrimp larvae (Fig. 1B–D).

Natural products have few to no side-effects and may thus represent a good alternative to current treatments for cocaine toxicity. In order to investigate whether plant-derived natural products can alleviate cocaine toxicity, we evaluated the seed extract of MT (containing 80% silymarin) on astroglial cells, the most abundant neuronal support cell in the adult brain (26,27). Death of astrocytes due to cocaine toxicity can lead to neuronal dysfunction. This problem can be largely avoided at the initial stages of cocaine addiction if astrocytes are protected from cocaine toxicity with compounds that are devoid of side-effects.

Our study demonstrated that MT has an antioxidant activity (Fig. 2A) and is non-toxic to C6 astroglial cells (Fig. 2B). When the cells were pretreated with MT extract for 30 min, followed by co-treatment with cocaine for 1 h, MT not only significantly sustained cell viability (Fig. 3A), but also contributed to the maintenance of cell morphological features (Fig. 3B), since MT-pretereated cells were similar to the control cells without vacuoles (Fig. 3C). Similar observations were recently reported for NAC pretreatment (13). In addition, we showed that the increased cell viability of cocaine-treated cells is accompanied by an increase in the GSH level. In order to investigate whether the increased cell viability is due to increased GSH levels with MT pretreatment, we measured the total GSH level in MT-pretreated cells.

The results of this experiment revealed that cocaine treatment causes a significant reduction in the total GSH level of the cells; however, pretreatment of cells with MT, followed by co-treatment with cocaine, significantly increased the GSH level (Fig. 4). Similar results were reported in C6 cells for cocaine and NAC treatment (13). The decrease in the GSH level in cocaine-treated cells and the increase in GSH due to pretreatment with either NAC (13) or MT (Fig. 4) suggest that compounds that induce intracellular GSH synthesis could be therapeutically effective against cocaine toxicity. The dose-dependent increase in the GSH level at 2, 3 and 4 mM cocaine-co-treated cells suggests that a different pathway may be responsible for the increase in the GSH level. Further studies will be needed to identify this mechanism.

NAC, an antioxidant compound, has been recognized as a relevant therapeutic agent for several oxidant-related CNS diseases (28). This compound was also proposed as a promising pharmacological agent for the treatment of cocaine dependence (29). Its administration to addicted individuals resulted in reduced cocaine-craving behavior (29,30). Since NAC hydrolysis in the body provides cysteine for intracellular GSH biosynthesis, it is possible that the observed benefit of NAC in cocaine addicts is related to the increased GSH level. No studies to date have provided evidence for an association between the increased GSH level and reduced cocaine-craving behavior in addicted individuals. If this is the case, then the role of additional natural products that support GSH synthesis in CNS cells and are devoid of serious side-effects merits further investigation. We argue that studies on natural products such as MT will provide alternative medicine for drug-induced psychiatric illnesses at a low price, and may reduce cocaine-related mental disorders such as depression, aggressiveness, and paranoia (31), possibly through an increase in the GSH level. Additional research is needed to explore this hypothesis.

## Abbreviations

BBB: blood brain barrier
CNS: central nervous system
DTNB: 5,5-dithiobis-2-nitrobenzoic acid
ED_50_: effective concentration needed to show 50% of a defined response
EDTA: ethylene diamine tetraacetic acid
FBS: fetal bovine serum
GSH: glutathione (total, i.e., oxidized and reduced)
LC_50_: lethal concentration needed to kill 50%of the cells
MT: milk thistle
NAC: N-acetyl-L-cysteine
NADPH: nicotinamide adenosine dinucleotide phosphate
OD: optical density
PBS: phosphate-buffered saline
